# In Vitro and In Vivo Phenotypes of Venezuelan, Eastern and Western Equine Encephalitis Viruses Derived from cDNA Clones of Human Isolates

**DOI:** 10.3390/v15010005

**Published:** 2022-12-20

**Authors:** Christina L. Gardner, Chengqun Sun, Matthew D. Dunn, Theron C. Gilliland, Derek W. Trobaugh, Yutaka Terada, Douglas S. Reed, Amy L. Hartman, William B. Klimstra

**Affiliations:** 1 Virology Division, U.S. Army Medical Research Institute of Infectious Diseases, Fort Detrick, MD 21702, USA; 2 The Center for Vaccine Research and Department of Immunology, The University of Pittsburgh, Pittsburgh, PA 15261, USA; 3 Elanco Animal Health, Greenfield, IN 46140, USA; 4 The Center for Vaccine Research and Department of Infectious Diseases and Microbiology, Graduate School of Public Health, The University of Pittsburgh, Pittsburgh, PA 15261, USA

**Keywords:** cDNA clone, equine, encephalitis, alphavirus, wild type, human isolate, FDA

## Abstract

The Department of Defense recently began an effort to improve and standardize virus challenge materials and efficacy determination strategies for testing therapeutics and vaccines. This includes stabilization of virus genome sequences in cDNA form where appropriate, use of human-derived virus isolates, and noninvasive strategies for determination of challenge virus replication. Eventually, it is desired that these approaches will satisfy the FDA “Animal Rule” for licensure, which substitutes animal efficacy data when human data are unlikely to be available. To this end, we created and examined the virulence phenotype of cDNA clones of prototypic human infection-derived strains of the alphaviruses, Venezuelan (VEEV INH9813), eastern (EEEV V105) and western (WEEV Fleming) equine encephalitis viruses, and created fluorescent and luminescent reporter expression vectors for evaluation of replication characteristics in vitro and in vivo. Sequences of minimally passaged isolates of each virus were used to synthesize full-length cDNA clones along with a T7 transcription promoter-based bacterial propagation vector. Viruses generated from the cDNA clones were compared with other “wild type” strains derived from cDNA clones and GenBank sequences to identify and eliminate putative tissue culture artifacts accumulated in the cell passaged biological stocks. This was followed by examination of aerosol and subcutaneous infection and disease in mouse models. A mutation that increased heparan sulfate binding was identified in the VEEV INH9813 biological isolate sequence and eliminated from the cDNA clone. Viruses derived from the new human isolate cDNA clones showed similar mouse virulence to existing clone-derived viruses after aerosol or subcutaneous inoculation.

## 1. Author Summary

Given the small numbers of human cases, it is most likely that FDA approval of vaccines and therapeutics targeting encephalitic alphaviruses will occur under the “Animal Rule” and be based primarily on efficacy studies performed in relevant animal models. To support this effort, we created cDNA clones that allow generation of uniform virus stocks for animal challenge that should be invariant between laboratories and over time. We used the sequences of human-derived isolates of Venezuelan, eastern and western equine encephalitis viruses, which were obtained from low passage cell-amplified virus isolates. To attempt to minimize the effect of cell-adaptive, presumably attenuating, mutations in clones derived from these sequences, we attempted to identify such mutations and reverted them to the type-specific residue for each virus. We compared the in vitro heparan sulfate binding characteristics of viruses derived from the human isolate cDNA clones, virulence in animals from subcutaneous or aerosol infection and growth rates of reporter expression vectors with those derived from cDNA clones that are well-represented in previous publications. The results of these studies provide human isolate cDNA clones for use in encephalitic alphavirus animal model challenge studies.

## 2. Introduction

Venezuelan, eastern and western equine encephalitis viruses (VEEV/EEEV/WEEV, respectively) are separate types of encephalitis-causing New World alphaviruses. Alphaviruses are small, enveloped RNA viruses that are naturally spread through the bite of an infected mosquito, which also have the potential for aerosol delivery during biowarfare [1]. In nature, VEEV circulates as six serological subtypes with different horse and human disease potential. Subtypes IA/B and IC show epizootic potential and are virulent for humans and horses, and are designated as Select Agents [2]. EEEV is found as two distinct subtypes. North American EEEV (NA EEEV) strains are highly virulent Select Agent viruses while South American strains (now Madariaga virus) are not designated Select Agents and exhibit less virulence, although severe human disease is seen in some outbreaks [3]. WEEV does not exhibit distinct serological subtypes and is not designated a Select Agent (reviewed in [4]). NA EEEV is one of the most acutely virulent viruses endemic to the Americas, causing mortality in 30–70% of symptomatic cases and similar rates of neurological sequelae in survivors, while VEEV and WEEV cause similar but lower levels of mortality in humans (~0.1–1% mortality for VEEV; ≥3% for WEEV) [5,6,7,8]. No licensed antivirals or vaccines are currently available for any of these viruses. As part of the U.S. Department of Defense efforts to protect the warfighter and civilians from possible exposure, animal models of disease after either subcutaneous or aerosol infection continue to be developed and refined for testing of antiviral therapeutics and vaccines [9].

The encephalitic alphaviruses are studied commonly in the adult mouse model since all three viruses cause brain disease and mortality after either subcutaneous (sc.) or aerosol infection (reviewed in [8]). WT VEEV epizootic strains generally cause uniform mortality by 5–8 days post sc. infection even at very low virion doses, while aerosol infection with similar doses slightly shortens survival time [10,11,12,13,14]. While survival times after sc. infection with EEEV are only slightly extended versus VEEV, a small percentage of mice occasionally survive sc. EEEV infection even at doses of ≥1000 PFU [15,16,17,18]. Similar sc. infections with WEEV yield further extended survival times and only produce ~60–70% mortality [19] (W.B.K. unpublished data). When given via aerosol, the virulence of both EEEV and WEEV is enhanced and “wild type” strains cause uniform mortality at common doses ([19,20] 2015).

As with biological stocks of all RNA viruses, alphaviruses undergo adaptation to the cultured cells upon which they are amplified. This process can be quite rapid as cell attachment phenotypes and virulence properties can be changed within the first three passages on cultured cells [21]. Therefore, the similarity of the genotypes/phenotypes of cell-amplified stocks to viruses circulating in nature is not clear unless direct sequence comparisons are made with unpassaged field samples [16]. Furthermore, the phenotypes of biological stocks potentially can change with time/cell passage and provide inconsistent results in animal challenge studies [22]. In contrast, viruses derived from cDNA clones should be minimally variable, depending upon the cell type used for production, and do not exhibit genetic change over time.

In an effort to standardize encephalitic alphavirus strains for production of challenge stocks to be used in antiviral and vaccine efficacy studies, we created cDNA clones from a low cell culture passage biological strain of each virus (VEEV IC INH9813, EEEV NA V105, WEEV Fleming) that was originally isolated from a human infection [23,24,25,26,27,28]. Full-length clones were synthesized directly from virus and vector sequences, avoiding any cloning process-based alteration of sequences (as has been noted with the VEEV 3000 TrD clone [10]). Well-characterized, human-derived isolates are preferred for pre-clinical alphavirus studies evaluating vaccine and antiviral therapeutic efficacy, which is likely to use mice and non-human primate models for final pre-clinical testing in accordance with the FDA “Animal Rule” [9]. Furthermore, we compared the mouse virulence of these viruses with strains derived from existing cDNA clones thought to represent low passage biological viruses isolated either from a donkey brain (VEEV IA/B Trinidad Donkey, [10,29]) a *Culiseta melanura* mosquito pool (NA EEEV FL93-939, Ref. [15]) or human blood (WEEV McMillan, [19,30]). The McMillan strain, while derived from an infected human, was passaged in adult mice during isolation [19] and, therefore, might represent a mouse-adapted virus.

## 3. Materials and Methods

### 3.1. Ethics Statement

All animal procedures were carried out under approval of the Institutional Animal Care and Use Committee of the University of Pittsburgh in protocols 15066059 and 18073259. Animal care and use were performed in accordance with the recommendations in the Guide for the Care and Use of Laboratory Animals of the National Research Council. Approved euthanasia criteria were based on weight loss and morbidity.

### 3.2. Cell Culture

BHK-21 (ATCC) were maintained in RPMI supplemented with 10% donor bovine serum (DBS), 10% tryptose-phosphate broth (TPB), 10,000 units/mL penicillin and 10 mg/mL streptomycin. CHO cells were maintained in Ham’s F12 medium with 10% fetal bovine serum (FBS) and L-Glutamine. Vero cells were maintained in DMEM-high glucose with 10% FBS, Pen/Strep and L-Glutamine as above. All cells were incubated at 37 °C in a 5% Co_2_ atmosphere.

### 3.3. cDNA Clone Sequences

Sequencing of biological isolates for construction of the VEEV V3000 (Trinidad Donkey strain a gift from Robert Johnston, UNC-Chapel Hill), EEEV FL93-939 (a gift from Scott Weaver, UTMB Galveston) and WEEV McMillan (a gift from Kenneth Olson, Colorado State University) cDNA clones has been published [10,30,31]. We used the GenBank sequences of the VEEV INH9813 (GenBank KP282671) and EEEV V105 (GenBank KP282670) and WEEV Fleming (GenBank MN477208) strains. Prior to GenBank submission, a Fleming sequence was a generous gift from Pamela Glass, USAMRIID.

### 3.4. cDNA Clone Construction and Virus Generation

**Figure 1:**
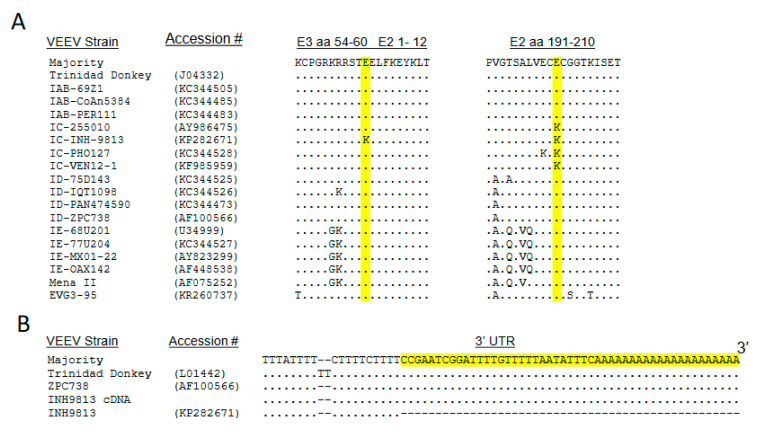
(**A**) Alignment of selected regions of the E3/E2 proteins of various VEEV epizootic and enzootic strains highlighting the E2 3 and E2 201 positive charge residues in the sequence of VEEV INH-9813. (**B**) Alignment of 3′ UTR sequences of various VEEV isolates and INH9813. The yellow highlighted sequence was added to the INH9813 cDNA clone.

**Figure 2:**
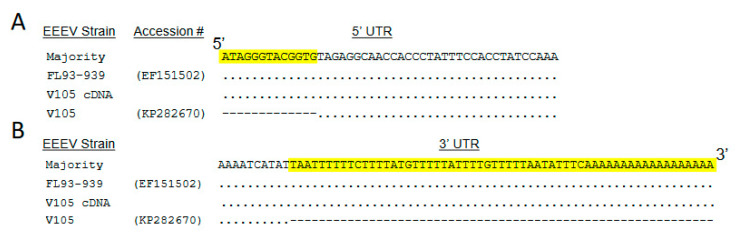
Alignment of selected regions of the 5‘ UTR (**A**) and 3′ UTR (**B**) showing nucleotide sequence information missing from the GenBank V105 sequence (yellow highlight) and sequence from FL93-939 used for the V105 cDNA clone. (**A**) Only 2 of 88 North American EEEV 5′ UTR sequences in GenBank had any differences with FL93-939 in the added region. (**B**) The added sequence is identical for 17 sequenced NA isolates including FL93-939.

The cDNA clones of Sindbis virus strain TR339 and 39H55K70 have been previously described [32,33]. For the VEEV INH9813 (GenBank KP282671), EEEV V105 (GenBank KP282670) [27] and WEEV Fleming (MN477208) virus strains, sequence data were compared with type-specific sequences available on GenBank. Sequence alignments were performed with MegAlign (DNAStar Inc. Madison, Wisconsin, USA). For VEEV, comparison was made with subtype IC strains only; for EEEV, comparison was with North American isolates only; and for WEEV, comparison was with available strains. The V105 cDNA sequence was then adjusted to match type-specific consensus sequences in the 5′ untranslated region (UTR) that were missing from the strain sequence (5′ 13nt identical in 86 of 88 NA EEEV GenBank sequences). Sequences of VEEV INH9813 and EEEV V105 were also missing the 3′ genome terminus; however, type-specific sequences in this area were not available for VEEV. Therefore, 27 nt of the TrD epizootic IA/B strain (identical to the enzootic ZPC738 ID strain) was added to INH9813 and 41 nt of the FL93-939 strain (EF151502; identical to 17 other EEEV NA strains) was added to V105 to complete the cDNA genomes (Figure 1 and Figure 2, respectively). The Fleming 3′ terminal poly adenlylation tract was set to 21 residues for each virus (as with the TrD cDNA [10]). The non type-specific E2 aa 3 lysine residue in the INH9813 sequence was retained for initial clone production. After initial testing, this residue was changed to the type-specific E2 aa 3 glutamic acid using Quick-Change PCR mutagenesis kit (Agilent, Santa Clara, CA, USA). The Fleming E2 valine and 84 lysine were also changed to the consensus 83 methionine and 84 glutamic acid by Quick Change PCR mutagenesis. The entirety of the E2 genes of mutagenized clones were sequenced to confirm changes. Full-length virus sequences were synthesized by Epoch Life Science including a pBR322-derived Amp^r^ plasmid, T7 promoter and linearization site (NotI) based upon the V3000 TrD cDNA clone [10]. The McMillan cDNA was altered by transferring the entire virus sequence from the previously published bacterial vector [30] into the modified pBR322 vector and T7 transcriptional promoter used here. This increased yields of viral RNA during in vitro transcription reactions.

Virus genomic RNA was generated from linearized cDNA templates by in vitro RNA synthesis (mMessage Machine, Invitrogen, Waltham, MA, USA) followed by electroporation of BHK cells and harvesting of supernatants at 24 h post infection (as previously described [34]). Supernatants were clarified by centrifugation, followed by use to infect Vero cells at an MOI of ~10 in a roller bottle culture and harvesting of supernatants at 24 h.p.i. Vero supernatants were clarified as above and subjected to discontinuous 20%/60% sucrose gradient centrifugation followed by pelleting, both at 24 krpm, and resuspending in OPTIMEM medium for storage and use in infections (as described [34]). All virus stocks were titered by standard BHK cell plaque assay.

### 3.5. Mouse Infections

Four-week-old female CD-1 mice were infected with each virus, either subcutaneously with either 1000 PFU in the left rear footpad, or in various doses via aerosol as described in individual experiments. Mouse experiments included groups of 5–10 mice. Subcutaneous infections were performed at least twice with similar results and aerosol LD_50_ infections were replicated with at least 4 dilutions per experiment. For aerosol exposures, mice were placed in a whole-body inhalation chamber within a class III biological safety cabinet maintained under negative pressure for 10 min to an aerosol containing the agent (pathogen) created by a 3-jet Collison nebulizer (CH Technologies, Westwood, NJ, USA) controlled by the AeroMP bioaerosol exposure system (Biaera Technologies, Hagerstown, MD, USA). The nebulizer creates a monodisperse aerosol between 1–2 µm in size, which will deposit in the deep lung. Aerosol concentration of the agent is determined by constant sampling of the chamber using an all-glass impinger (AGI; Ace Glass, Vineland, NJ, USA). Inhaled (presented) dose was determined as the product of the aerosol concentration of the virus, the duration of the exposure, and murine minute volume as calculated by Guyton’s formula. Mice were observed once daily until signs of infection were observed and twice daily thereafter. Morbidity and mortality scores were recorded for 21 days after infection. General clinical signs of infection were assessed by visual inspection, as well as daily weighing of the animals. The visual scoring system used was as follows: “0” for no disease signs; “1” for ruffled fur; “2” for mild behavioral abnormalities including altered mobility, lethargy; “3” for more severe behavioral disturbances including ataxia; “4” for paralysis or seizures. Animals that developed severe disease signs prognostic of eventual mortality, defined by >35% body weight loss or morbidity score of 4, were immediately euthanized.

### 3.6. Plaque Size Determination

Plaque sizes were measured during a standard BHK cell plaque assay using 0.5% immunodiffusion agarose (MP Biomedicals Irvine, CA, USA) for overlay. Plaque assay plates were stained with neutral (as described in [21]) followed by inversion over a light box and measurement of plaque diameters with a metric ruler. Sixty plaques across three wells were counted for each virus and each experiment was performed twice with similar results.

### 3.7. Relative Infectivity Assay

We infected 24-well plates of CHO-K1 and pgsA-745 cells with the indicated viruses for 1 h at 37 °C followed by 3-times washing with PBS and immunodiffusion agarose overlay as in plaque assays. At 24–48 h post-infection, cells were fixed with 4% paraformaldehyde and infection foci were counted on an inverted fluorescence microscope. For some viruses, mCherry or GFP expressing viruses were used. For other viruses, cells were stained for viral antigen with ATCC polyclonal antibody as previously described [35].

### 3.8. Expression of Infection Reporter Proteins

Each virus cDNA was derivatized by insertion of enhanced green fluorescent protein gene, mCherry gene or nanoLuciferase gene (nLuc; Promega) as an in-frame cleavable element between the capsid and PE2 genes using the *Thosea asigna* 2A-like protease for C-terminal cleavage (exactly as described in [33]). For nLuc growth analysis, BHK cells were infected at an MOI of 0.1 BHK PFU and harvested at various intervals p.i. in nLuc cell lysis buffer (Promega) followed by in vitro nLuc assay using the Promega nano-Glo kit.

### 3.9. Statistical Analysis

Mouse mortality profiles and growth curves were analyzed by Mantel–Cox Log Rank analysis and significance of plaque size differences and weight differences was determined by Student’s *t* test *(p* > 0.05 is considered non-significant). LD_50_ values were calculated by using nonlinear fit regression log (inhibitor) vs. response (three parameters) analysis. Determination of differences between LD_50_s of the viruses were determined by using nonlinear regression (inhibitor vs. normal response-variable slope analysis). All analysis was done using GraphPad (La Jolla, CA, USA) PRISM 9 software.

## 4. Results

### 4.1. Comparison of Biological Isolate Sequences with Type-Specific Sequences

The most accurate strategy for producing virus cDNA clones is to utilize RT-PCR amplification of unpassaged field samples followed by direct synthesis of cDNA clones, thus avoiding the acquisition of cell adaptive effects as described above. However, such sequences are not currently available for human isolates of the encephalitic alphaviruses. In the past, we have validated cDNA sequences by comparison with field isolate partial sequences when available [16]. In the current studies, sequencing of low passage cell culture-amplified strains was used as a basis for clone synthesis (Table 1). The EEEV V105 sequence on GenBank (KP282670) does not cover the extreme 5′ and 3′ untranslated regions (UTRs) of the genome. Therefore, nucleotides of North American type-specific GenBank consensus sequences were inserted in the EEEV cDNA clone as indicated in Figure 2A,B. Similarly, the INH9813 GenBank sequence (KP282671) does not cover the last segment of the 3′ UTR (Figure 1B), and nucleotide sequences for the region that was identical in multiple VEEV clades were added to the synthesized cDNA.

**Figure 3:**
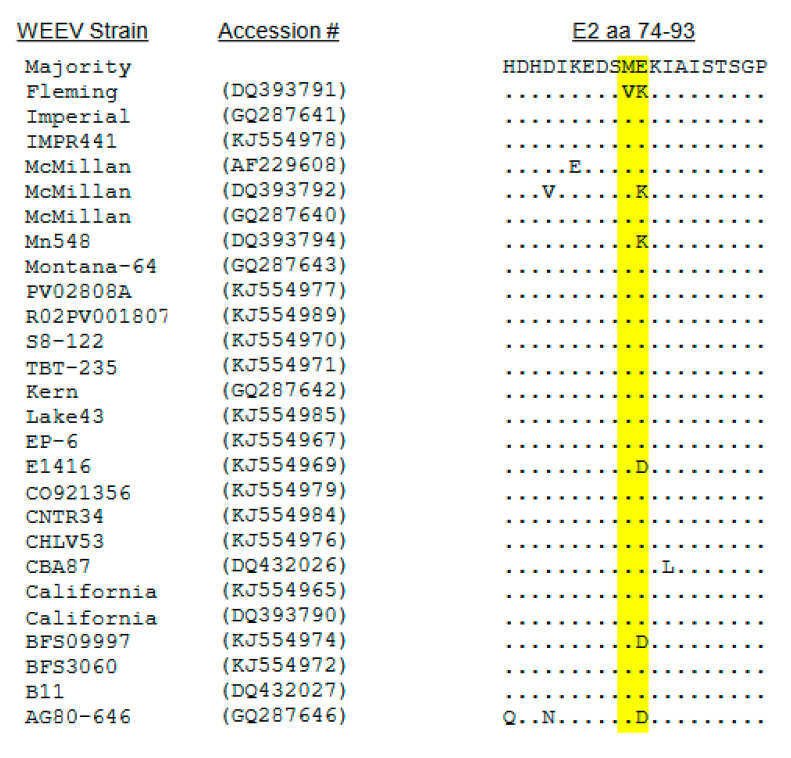
Alignment of the differences between WEEV Fleming and other WEEV strains in the E2 70 region illustrating amino acid sequence differences tested for effects upon HS dependent infectivity (yellow).

Regarding other genome regions, particular attention was paid to the presence of non type-specific positively charged amino acids in the translated E2 gene amino acid sequence of each sequenced human isolate as mutations to positively charged amino acids that increase binding to heparan sulfate (HS) can be rapidly selected upon passage of alphaviruses in cultured cells [21]. Furthermore, adaptive mutations can arise at different E2 positions in a single virus isolate depending upon the cell type used for passage and, potentially, other factors [36,37]. For VEEV INH9813, a change from glutamic acid (type-specific consensus) to lysine at position 3 of the E2 protein was observed (Figure 1). Positive charge substitution mutations in this position of VEEV E2 had previously been associated with enhanced HS binding or BHK cell passage [37,38]. In addition, an IC type-specific change from glutamic acid (VEEV consensus) to lysine was observed at E2 201 (Figure 1). For EEEV V105, no changes were observed in the E2 sequence compared to FL93-939 [15] or the consensus of NA EEEV strains sequenced directly from PCR-amplified primary field isolates without cell culture amplification [16,39]. Consequently, the V105 E2 sequence was considered “wild type” and directly reflective of naturally circulating viruses. For the WEEV Fleming E2 protein, valine and lysine were substituted for methionine and glutamic acid, respectively, at position s 83 and 84 versus the type consensus (Figure 3). However, two other WEEV strains, including one of several sequences for the McMillan isolate in GenBank, possessed lysine at E2 84 (Figure 3). The WEEV McMillan isolate cDNA used in our studies did not possess the lysine substitution at E2 84 [30].

### 4.2. In Vitro Phenotype Comparison of Viruses Derived from cDNA Clones

**Figure 4:**
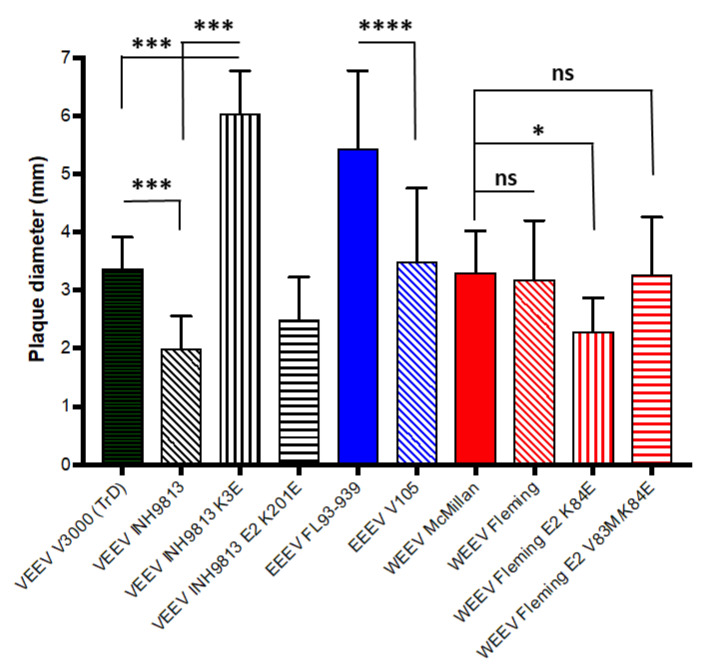
Plaque sizes of cDNA clone-derived viruses on BHK cells. Plaque assays were performed as described in Materials and Methods and 60 plaques in three wells were counted for each virus. VEEV—black bars, EEEV—blue bars, WEEV—red bars. Error bars are standard deviations. Significance denoted with asterisks or ns if not significant (ns *p* > 0.05, * *p* < 0.01, *** *p* < 0.0001, **** *p* < 0.00001).

Viruses were generated from each cDNA clone listed in Table 1 and typical BHK cell tiers after high efficiency electroporation of in vitro synthesized RNA was determined. Plaque sizes can differ between strains of alphaviruses [21] and multiple distinct plaque sizes can be observed in single stocks of biological viruses such as those sequenced for creation of the INH9813, V105 and Fleming clones [9]. We measured plaque sizes for BHK stocks derived from each human isolate clone and assessed them for uniformity of size on BHK cell monolayers and compared them with viruses derived from the other VEEV, EEEV and WEEV isolate cDNA clones (Figure 4). With VEEV, INH9813 plaque sizes were significantly smaller than those of TrD (*p* < 0.001). Plaque sizes were also significantly different comparing EEEV V105 and FL93–939 (*p* < 0.001). However, plaque sizes for WEEV Fleming and McMillan were not significantly different (*p* = 0.470).

**Figure 5:**
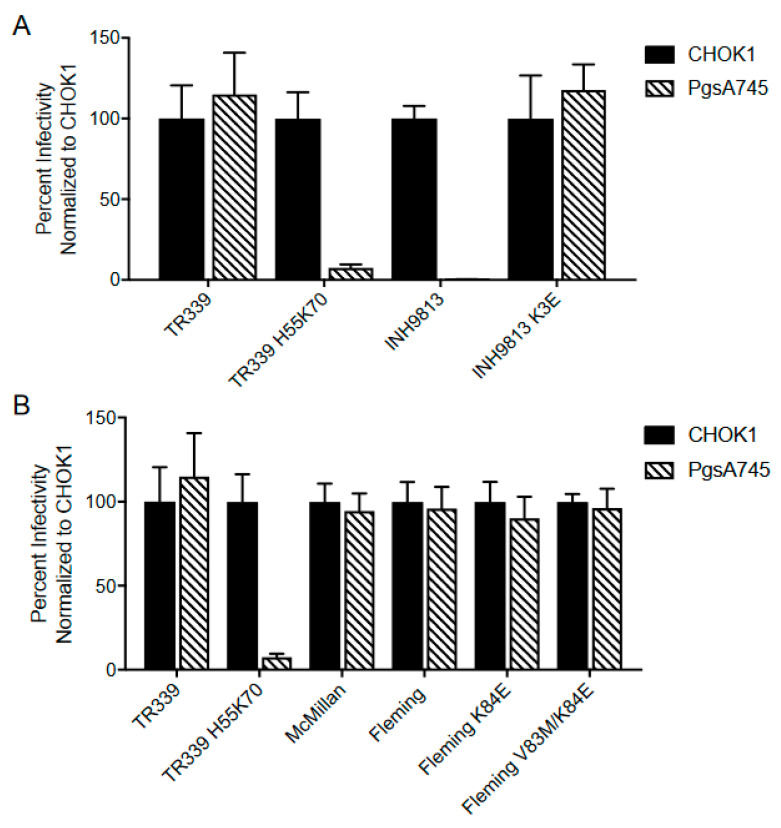
Relative infectivity of the indicated VEEV (**A**) and WEEV (**B**) viruses on WT CHOK1 and GAG-deficient pgsA745 cells. SINV TR339 and H55K70 are included as HS–independent and HS–dependent controls, respectively. GFP or mCherry expressing foci were enumerated with a fluorescence microscope. Infectivity was measured as the percentage of WT infectious units observed on each cell type with WT CHO cells set to 100%. Error bars are standard deviations and some are too small to be seen.

We examined the effect of the VEEV INH9813 E2 3 lysine and E2 201 lysine and the WEEV Fleming E2 83 valine and E2 84 lysine differences by constructing clones with the consensus amino acids at these positions and then examining the dependence of cultured cell infectivity upon cell surface HS. Reversion of INH9813 E2 3 lysine to the type-specific glutamic acid significantly increased plaque sizes versus INH9813 (*p* < 0.001) with plaques for the resultant virus also significantly greater than V3000 (*p* < 0.001). The lysine at E2 3 also conferred significantly higher dependence upon HS for infection versus the type-specific residue evidenced by reduced infectivity for HS-deficient CHO pgsA–745 cells (Figure 5A) (*p* < 0.001). VEEV consensus-specific reversion of the E2 201 lysine did slightly and significantly affect plaque size (*p* < 0.001). We did not examine HS dependence for this virus as the lysine at 201 as it had previously been associated with virulent epizootic IC strain emergence [37]. Lysine was found at this position in all sequenced IC strains and mutation to the glutamic acid residue was attenuating in mice (see below). These data suggest that the 201 lysine is important for virulence of IC viruses. With WEEV Fleming, neither the E2 84 lysine nor this mutation combined with E2 83 valine affected the HS dependence of infectivity (*p* > 0.05), although the E2 84 lysine alone did reduce plaque sizes slightly (*p* < 0.010) (Figure 4 and Figure 5B). From these data, we conclude that the INH9813 E2 3 lysine residue represents a cell-adaptive mutation that increases HS binding concomitant with a large impact upon plaque sizes on BHK cells.

### 4.3. In Vivo Phenotype Comparison of Viruses Derived from cDNA Clones

**Figure 6:**
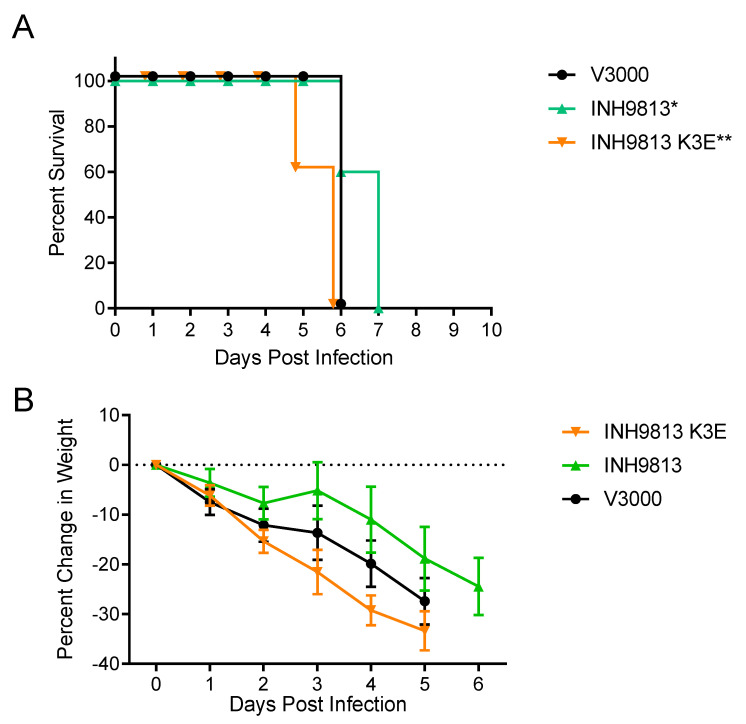
Survival curves (**A**) and weight loss (**B**) for 4–week–old CD–1 mice infected subcutaneously with 1000 PFU of each indicated virus. Experiments included 5 or 10 animals per group and were performed at least twice with similar results. Error bars are standard deviations. * = Survival V3000 vs. INH9813 *p* < 0.001, ** = Survival INH9813 vs. INH9813 K3E *p* < 0.001.

**Figure 7:**
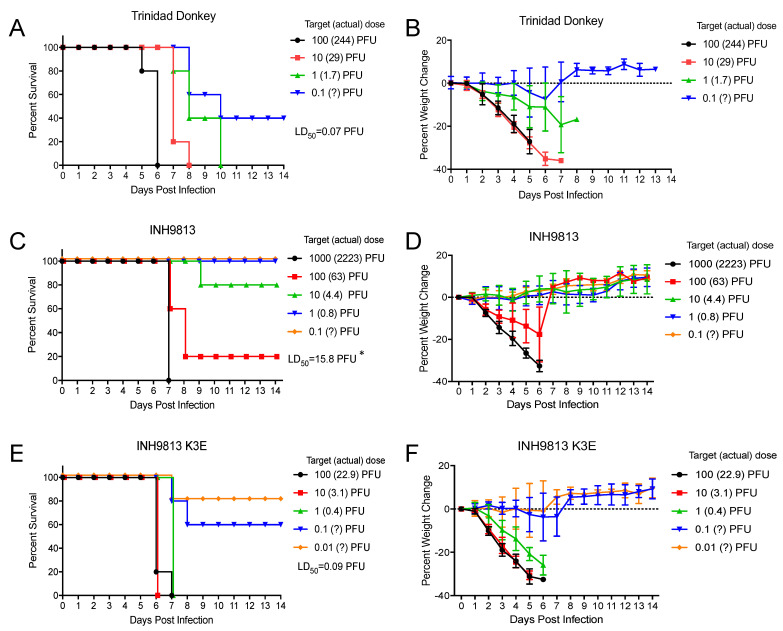
Aerosol LD_50_ mortality curves (**A**,**C**,**E**) and weight loss data (**B**,**D**,**F**) for 4–week–old CD–1 mice infected with each indicated virus. Values with question marks are estimates of aerosol dose. Aerosol LD_50_ infections included 5 or 10 mice per group and were replicated with at least four dilutions per experiment. Error bars are standard deviations. * = LD_50_ V3000 vs. INH9813 *p* < 0.002.

The four-week-old CD-1 female mouse is very sensitive to subcutaneous (sc.) or aerosol infection with VEEV, EEEV and WEEV, and can be used to distinguish subtle differences in virulence [16,40,41] and was chosen for comparison experiments. Mice were either infected subcutaneously (sc.) with 1000 PFU of each virus or with a series of virus dilutions for determination of an aerosol dose LD_50_. After sc. inoculation, the VEEV virus INH9813 with lysine at position 3 of the E2 glycoprotein showed a significantly milder disease compared with the virus derived from the V3000 cDNA (*p* < 0.001) (Figure 6A). This included a one-day increase in survival time and slower weight loss (Figure 6A,B). Importantly, INH9813 was significantly less virulent than the virus with the E2 residue changed to the type-specific INH9813 glutamic acid (K3E) (*p* < 0.001). However, when the lysine at E2 3 was reverted to the IC type-specific glutamic acid, survival times were slightly less than V3000 (*p* = 0.029). Calculated LD_50_ values from aerosol infections comparing V3000 and INH9813 also indicated that the latter was less virulent (Figure 7). The estimated LD_50_ (estimated due to the fact that the V3000 LD_50_ was <1 BHK PFU) for V3000 was 0.07 PFU while it was 15.8 PFU for INH9813 (a significant difference, *p* < 0.002), and when the lysine at 3 was reverted to glutamic acid, the INH9813 K3E LD_50_ was 0.09 PFU (*p* > 0.05 versus V3000).

**Figure 8:**
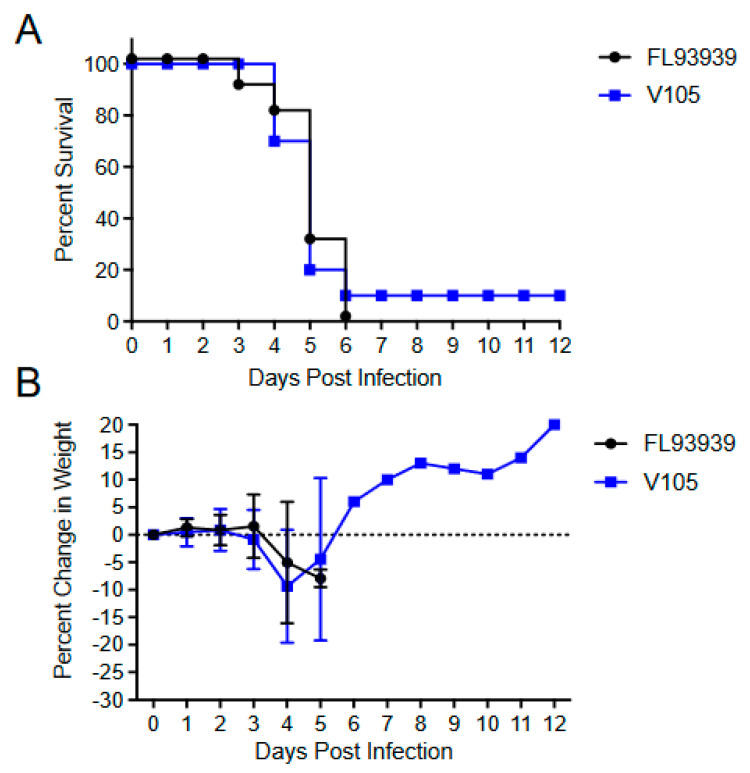
Survival curves (**A**) and weight loss (**B**) for 4–week–old CD–1 mice infected subcutaneously with 1000 PFU of each indicated virus. Experiments included 5 or 10 animals per group and were performed at least twice with similar results. Error bars are standard deviations. LD_50_ Fl93–933 vs. V105 not significantly different.

**Figure 9:**
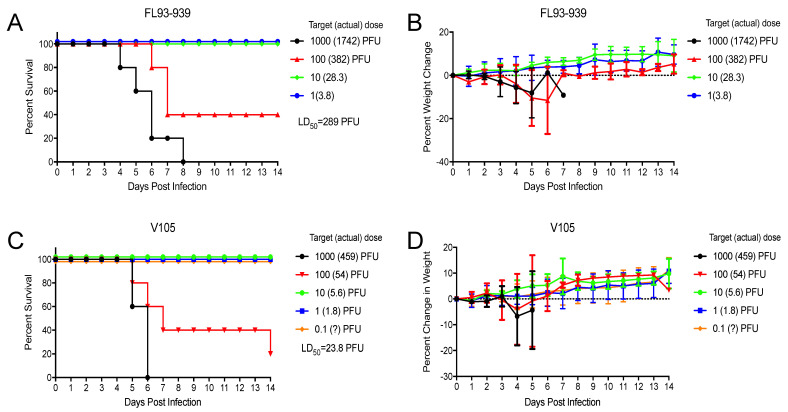
Aerosol LD_50_ mortality curves (**A**,**C**) and weight loss data (**B**,**D**) for 4–week–old CD–1 mice infected with each indicated virus. Values with question marks are estimates of aerosol dose based on virus dilution. Aerosol LD_50_ infections included 5 or 10 mice per group and were replicated with at least four dilutions per experiment. Error bars are standard deviations.

With EEEV, sc. infection survival times for EEEV V105 and FL93–939 were not significantly different (*p* > 0.916) (Figure 8A). However, V105 infections in this experiment series did yield one survivor (Figure 8A), which is also occasionally seen with FL93–939. Aerosol LD_50_ values were also not significantly higher for FL93–939 vs. V105 (*p* > 0.05) (Figure 9), although calculated values were lower for V105.

**Figure 10:**
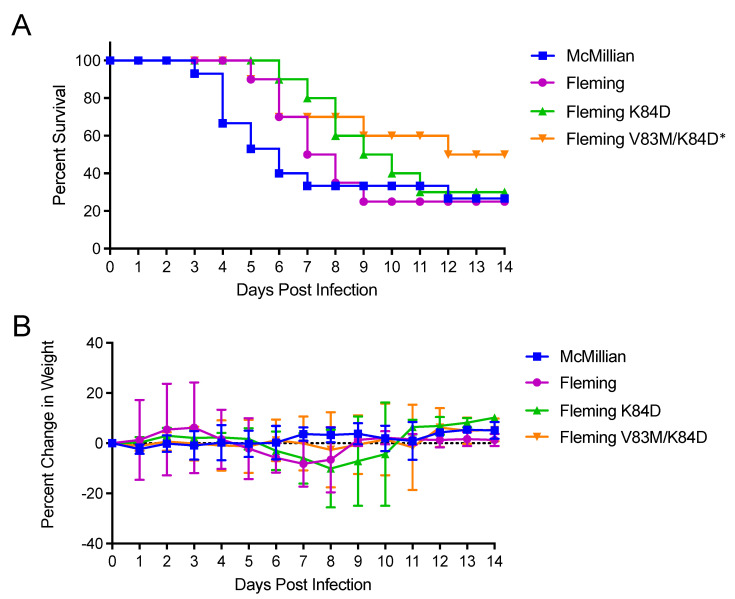
Survival curves (**A**) and weight loss (**B**) for 4–week–old CD–1 mice infected subcutaneously with 1000 PFU of each indicated virus. Experiments included 5 or 10 animals per group and were performed at least twice with similar results. Error bars are standard deviations. * = Survival Fleming vs. Fleming V83M/K84D *p* = 0.025.

**Figure 11:**
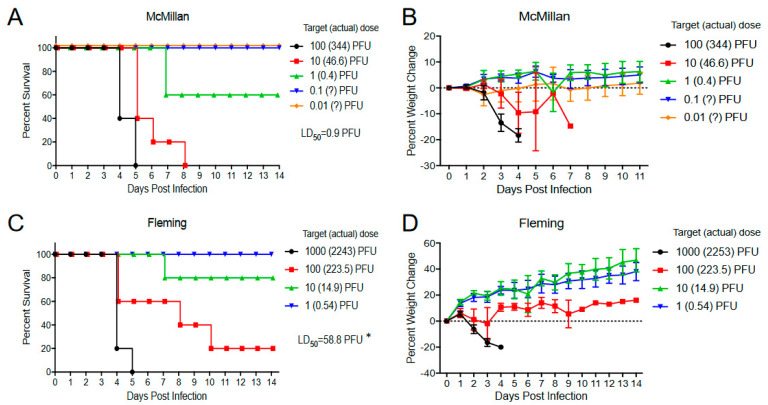
Aerosol LD_50_ mortality curves (**A**,**C**) and weight loss data (**B**,**D**) for 4–week–old CD–1 mice infected with each indicated virus. Values with question marks are estimates of aerosol dose based on virus dilution. Aerosol LD_50_ infections included 5 or 10 mice per group and were replicated with at least four dilutions per experiment. Error bars are standard deviations. * = LD_50_ McMillian vs. Fleming *p* < 0.001.

With WEEV, we compared the McMillan clone with either Fleming or this virus with lysine changed to glutamic acid at E2 84 or this mutation and valine changed to methionine at E2 83 (K84D, V83M) (Figure 10). After sc. infection, McMillan and Fleming survival times were not significantly different (*p* > 0.436) and neither virus was significantly different from the single glutamic acid 84 mutant (K84D, *p* = 0.306 and *p* = 0.369, respectively) (Figure 10A). However, the double mutant was significantly attenuated versus Fleming (*p* = 0.025) (Figure 10A) and slightly but not significantly different from McMillan (*p* = 0.126). Interestingly, the McMillan aerosol LD_50_ was significantly lower than that of Fleming (0.9 versus 58.8 PFU, *p* < 0.001) (Figure 11). The E2 mutant WEEVs were not tested in aerosol infections due to attenuation observed after sc. infection.

### 4.4. Comparisons of Reporter Expressing Viruses

**Figure 12:**
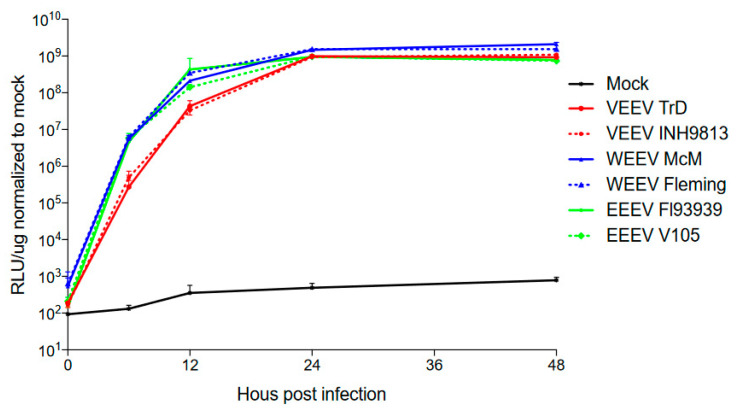
Time course of nanoLuciferase assay results in relative light units/μg of protein after infection of BHK cells with vectors derived from each indicated virus. All INH9813 viruses were the K3E “wild type” mutant. The MOI was 0.1 PFU.

To compare in vitro growth of the viruses, we examined the relative expression of nanoLuciferase (nLuc) from viruses expressing the reporters as cleavable fusions between the capsid and PE2 genes (as in [31]) by infecting BHK cells at an MOI of 0.1 BHK PFU and then performing nLuc assays on lysates (Figure 12). Expression curves were not significantly different within virus types (TrD vs. INH9813 K3E, *p* = 0.1564, FL93 vs. V105, *p* = 0.1161, Fleming vs. McMillan, *p* = 0.0632). These results suggest that the clone-derived viruses for each type are similar in transgene expression and in vitro replication kinetics.

## 5. Discussion

Here, we have used an informed approach to analyze sequences derived from biological virus stocks and created cDNA clones from human isolates of VEEV (INH9813), EEEV (V105) and WEEV (Fleming). Missing deep sequence information for the extreme 3′ U’TR of VEEV INH9813 and from the 5′ and 3′ UTRs of EEEV V105 sequence was added from a consensus of sequences available in GenBank. In addition, a deletion in the nsP 4 region of WEEV Fleming was re-created using consensus amino acid sequence derived using the McMillan codon preference. However, subsequent to creation of the Fleming cDNA clone, a revised sequence of Fleming was published [42]. The sequence in the area of the deletion was identical to our inserted sequence. Finally, E2 glycoprotein amino acid sequences were compared to the type-specific consensus for each strain and positive charge substitutions in the individual strain sequences were tested for their effects upon virulence and HS-dependent infectivity. This was followed by comparisons of mouse virulence with viruses derived from existing cDNA clones, each virus constructed by other groups [10,15,30]. It is anticipated that virus stocks derived from the new human isolate clones could be used as challenge material to satisfy the FDA “Animal Rule” during pre-clinical testing of anti-alphavirus vaccines and therapeutics [9]. We recently published a detailed examination of the disease phenotypes of the VEEV and EEEV cDNA-derived viruses in the cynomolgus macaque model for these purposes [34,43,44,45]. Furthermore, in the macaque studies, we sequenced the genome of NHP stock virus generated from the INH9813 clone and the sequences were identical, suggesting that our stock production conditions do not alter genotypes [34].

We compared the sc. weight loss and mortality and aerosol infection LD_50_ values after infection of adult CD-1 mice between the new human isolate-cloned viruses and existing clone-derived viruses. With VEEV, the INH9813 IC strain was compared with the V3000 clone of the Trinidad Donkey IA/B strain. Both of these are epizootic human-virulent viruses; however, their comparative virulence has not been determined with cDNA clone-derived viruses. Comparisons of mouse virulence of cDNA-generated viruses with biological progenitor strains (V3000 Trd Clone [10]; FL93-939 [15]; and WEEV Fleming [30]) suggested virulence similar to biological viruses. Furthermore, there were not significant differences in sc. virulence (AST or weight loss) or aerosol LD_50_ in mice between different strain cDNA clones of EEEV or between IA/B, or IC isolates of VEEV. However, there was a trend towards lower aerosol LD_50_ for the EEEV V105 strain compared with FL93-939. With WEEV, the McMillan clone-derived virus exhibited a significantly lower aerosol LD_50_ than the Fleming strain and this difference should be investigated further, as both of these isolates were derived from human infections; as mentioned previously, the McMillan biological isolate was subjected to adult mouse passage. Similarly, in macaques, McM is capable of uniform lethality after aerosol infection but Fleming is not (Klimstra, Hartman, Reed unpublished data).

Our results support the longstanding hypothesis that even low-passage biological viruses amplified in vitro can possess mutations that enhance replication in cultured cells, in particular mutations in the E2 glycoprotein that increase infection dependence upon HS [21] (reviewed in [46]). These have been shown to arise very rapidly in experimental settings that simulate biological virus amplification [21]. Furthermore, these changes are generally attenuating to alphavirus disease in vivo (current studies) [21,32,38] and can affect plaque size in vitro (current studies) [21,32,47,48]. Absent the type of unpassaged field isolate sequence data available for EEEV [16,39], it cannot be unequivocally stated that positive charge mutations in individual alphavirus stocks that confer enhanced HS dependent infectivity on CHOK1 cells are tissue culture-adaptive variations in naturally circulating strains as opposed to natural variation between isolates (as we have observed with EEEV, [49]). However, if reversion of these variations to the type-specific consensus significantly increases virulence in animal models, as was the case with VEEV INH9813 E2 3 lysine, it is most likely that they arose during in vitro amplification. We therefore consider the INH9813 K3E mutant to be “wild type” INH9813.

Since only the VEEV INH9813 biological stock possessed a non type-specific E2 variant that increased HS dependence in our assays, it is clear that in vitro selection pressures affect virus stocks differently and cell-adaptive mutations may not be present in some low passage stocks. For example, we have not seen the acquisition of positive charge mutations during repeated passage of EEEV stocks, most likely reflecting the efficient interaction of naturally circulating strains virus with HS [16] such that mutations would not greatly increase infectivity for cultured cells. However, we also did not detect non type-specific positive-charge mutations that increased HS-dependent infectivity in sequences of the WEEV Fleming or the McMillan cDNA clones. Notably, sequences of different stocks of the McMillan isolate in the GenBank database did differ by positive charge substitutions in E2, but these, when tested, did not significantly alter the HS dependence of infectivity, at least with CHO cell infection. Furthermore, we have not considered the possibility of mutations that might occur in genome regions other than the E2 protein. Ultimately, until primary field isolate tissues are directly sequenced followed by resurrection in cDNA clone form, the genotype of alphavirus strains cannot be determined unequivocally.

An argument could be made that quasispecies diversity, which has been linked to in vivo replicative fitness and virulence for RNA viruses (reviewed in [50,51,52]), could be suppressed by derivation of viruses from cDNA clones, which may yield relatively homogenous virus populations. However, with cell culture-passaged virus stocks, the quasispecies would be determined during replication in cultured cells and enriched in variants selected for replication in cell cultures, typically fibroblastic cells. This would likely be quite different from quasispecies derived from tissues of infected vertebrate hosts/mosquitoes and, therefore, the relevance of such in vitro diversity is questionable. For the cDNA-derived viruses, we assume that some degree of quasispecies diversity is generated during the initial replication of the virus in the electroporated cells and, if electroporation supernatants are used for a single round amplification of stocks, this would be further enhanced. Furthermore, the disadvantage to in vivo fitness of acquisition of cell adaptive mutations in passaged strains, which can be quantitated in studies such as these and others [21,36,38,53,54,55] is very clear. Finally, a potentially equally important point is the fact that stocks of these mosquito-vectored viruses are commonly derived from vertebrate cells as opposed to mosquito vector cells, which occurs exclusively during natural infection. In contrast, the source of viruses that might be utilized in a malicious release is not clear. Ultimately, consideration of the intended use of the clone-derived virus stock should drive the choice of cells used for virus production and, possibly, the cDNA clone virus itself, with the caveat that protecting against the most animal-virulent form of a particular virus is likely the most prudent strategy.

The guidance that the FDA provides for the “Animal Rule” in regard to challenge material is that it should be an isolate shown to have caused human disease, that it should be well-characterized and have a low passage history [9]. The strains of VEEV, WEEV and EEEV selected here were originally isolated from human cases but had been passaged in cells and, in at least one case, a mutation was acquired that was attenuating to animal virulence. The cDNA clones we described here would meet FDA requirements while also providing a means to generate challenge material sufficient to support rigorously standardized efficacy studies of vaccines or therapeutics and better ensure comparability between these studies.

## Figures and Tables

**Table 1:** In vitro passage history of biological isolates used for cDNA clone construction.

Virus/Clone	Subtype	Passage ^1^	Reference
VEEV Trinidad Donkey/V3000	IA/B epizootic	GP-1, CE-14, SMB-1, V-2, and BHK-1	(P. Glass Pers. Comm.)
VEEV INH9813	IC epizootic	V-3	[26]
EEEV FL93-939	North American	V-1, SMB-1	[15]
EEEV V105	North American	V-3	[26]
WEEV McMillan	-	M-2, SMB-1, V-2	[19]
WEEV Fleming	-	SMB-5, V-3	(S. Weaver, Pers. Comm.)

^1^ M—mouse, SMB—suckling mouse brain, CE—chick embryo, GP—guinea pig, BHK—baby hamster kidney cell, V—Vero cell.

## Data Availability

All data is available upon request from the corresponding author.
